# Work-related musculoskeletal disorders among registered general nurses: a case of a large central hospital in Harare, Zimbabwe

**DOI:** 10.1186/s13104-018-3412-8

**Published:** 2018-05-18

**Authors:** M. Chiwaridzo, V. Makotore, J. M. Dambi, N. Munambah, M. Mhlanga

**Affiliations:** 1 Rehabilitation Department, University of Zimbabwe, College of Health Sciences, P.O Box A178, Avondale, Harare, Zimbabwe; 2 Howard Hospital, P.O Box 190, Glendale, Zimbabwe; 3 Department of Nursing Science, University of Zimbabwe, College of Health Sciences, P.O Box A178, Avondale, Harare, Zimbabwe

**Keywords:** Prevalence, Associated factors, Nurses, Parirenyatwa Group of Hospitals

## Abstract

**Objective:**

Worldwide, work-related musculoskeletal disorders (WMSDs) are a common cause of morbidity affecting occupational individuals such as health-care professionals. However, nothing is known about WMSDs in hospital nurses in Zimbabwe. This study was conducted to provide cross-sectional evidence of the 12-month prevalence, consequences and factors associated with WMSDs among 208 nurses at Parirenyatwa Group of Hospitals (PGH).

**Results:**

The response rate for the study was 55.7%. The median age for the participants was 32.0 years (interquartile range = 29–36 years). The lifetime prevalence of WMSDs in nurses was 95.7% (n = 112). The first episodes were experienced in the first 5 years of working (n = 59, 52.7%). However, 82.1% (n = 96) nurses experienced WMSDs in the last 12 months. Low back pain was the most common WMSDs reported (n = 55, 67.9%). WMSDs were significantly associated with qualification attained, postgraduate ergonomic training and working experience. Overall, 87.5% (n = 84) nurses experienced at least one of the consequences of WMSDs. Cognisant of the limitations of the study, the present study found that WMSDs are a common occurrence among nurses at PGH. This creates a need for prompt hospital education programs aimed at raising awareness among nurses on the existence of WMSDs and the consequences at PGH.

**Electronic supplementary material:**

The online version of this article (10.1186/s13104-018-3412-8) contains supplementary material, which is available to authorized users.

## Introduction

Work-related musculoskeletal disorders (WMSDs) are a common cause of morbidity affecting occupational people such as health professionals [1–9]. Nurses are reportedly the hardest hit among health-care professionals [10–15]. Unabated, WMSDs interfere with productive work and quality of life [1, 4, 11, 13, 16, 17]. The prevalence of WMSDs among nurses varies between studies. Tinubu et al. [7] reported a prevalence of 78% among Nigerian nurses, with WMSDs associated with working in the same positions for long periods, lifting/transferring of patients and increased patient load. Yan et al. [11] observed a prevalence of 77.4% among Chinese nurses. Two systematic reviews cited poor patient transfer techniques, physical nature of the job, excessive repetition, and awkward postures as factors associated with WMSDs [13, 18].

Currently, there is dearth of literature documenting the magnitude, consequences and factors associated with WMSDs in Zimbabwean nurses as compared to other health professionals [19]. Surely, this does not insinuate that Zimbabwean nurses are immune to WMSDs. Although Zimbabwean nurses are not dissimilar from other nurses, contextual differences necessitate local studies to be conducted for relevant solutions to be proffered. Understanding the prevalence and factors associated with WMSDs among nurses is important for health policy administrators and health-care workers to curtail the existence of the problem. Therefore, this study was conducted to determine the 12-month prevalence, consequences and factors associated with WMSDs among nurses at PGH.

## Main text

### Study design, research setting and participants

A cross-sectional study was conducted at PGH targeting registered general nurses (RGNs). This hospital was chosen because it is the largest public hospital in Zimbabwe. It has 35 wards and for the purposes of this study were divided into medical, surgical, maternity and others (critical units i.e. intensive care unit, coronary care unit, burns unit, theatre unit, outpatient department, and casualty unit). The following parameters were used to estimate sample size using EPI info StatCalc: (i) total population of nurses at PGH (N = 762) (ii) expected prevalence of 80.8% [20], (iii) precision effect of 5%, (iv) design effect of 1 and (v) expected non-response rate of 14.6% [20]. Stratified proportional random sampling was then used to select 208 nurses in the various wards. Nurses above 18 years of age and freely willing to participate were included. RGNs had to have at least 1 year working experience at PGH performing clinical duties. Pregnant nurses and those who had given birth in the last 3 months were excluded [12].

### Survey instrument

The questionnaire was largely researcher-developed and had few adopted questions from the Nordic Musculoskeletal Questionnaire (NMQ) [21–23]. The questionnaire was divided into four sections. The first section elicited information on socio-demographic and work-related information. Section A had questions on WMSDs and body regions affected. Section B elicited data on the consequences of WMSDs. Section C presented job tasks perceived to be associated with WMSDs. The questionnaire showed high Scale/Average Content Validity Index (S-CVI/Ave = 0.99) using the criteria outlined in literature [24, 25]. Thereafter, the questionnaire was translated into Shona following guidelines propounded by Sousa et al. [26]. The English questionnaire was evaluated for test–retest reliability among 30 nurses and showed kappa coefficients (k) ranging from 0.36 to 1, suggesting fair to perfect agreement according to Landis and Koch [27] (Additional file 1).

### Procedure

This study adhered to the ethical principles under the Declaration of Helsinki [28]. Only the participants who read the information letters and agreed to sign the informed consent form participated. The questionnaires were self-administered with the option of returning them immediately or later. Those who completed later were given a maximum of 7 days to return the questionnaires. Telephonic and SMS reminders were sent within the 7 days reminding participants to complete the questionnaires.

### Statistical analysis

Data was analysed using Statistica version 13.2. Normality for data was assessed using the Kolmogorov–Smirnov and Lilliefors test. Descriptive statistics were used to describe socio-demographic characteristics of the respondents. The Mann–Whitney U test checked for significant differences in the sum ranks of the ages by gender. Kruskal–Wallis One-way analysis of variance (ANOVA) checked for differences in the sum ranks in the years of experience for nurses by ward. Factors associated with WMSDs were evaluated using Chi square and the Fishers’ exact test (p ≤ 0.05).

## Results

The response rate for the study was 55.7% (n = 117). The sample socio-demographic and work-related data are shown in Table 1. Age data was not normally distributed (K-S d = 0.13, p < 0.05; Lilliefors p < 0.01). The median age for the participants was 32.0 years (interquartile range, IQR = 29–36 years). There was a significant difference in the rank sum of the ages by gender (U = 453, p = 0.001). The median years of working experience for the nurses was 7.0 (IQR = 3–10) years. There was a significant difference in the median years of working between the sexes [U = 454, p < 0.01]. Females had more experience than males. Nurses in the maternity wards had more years of experience than the rest [H (3, N = 117) = 27.5, p < 0.01].

**Table 1:** Sample characteristics (N = 117)

Variable	n (%)
Age (years)	
20–29	36 (30.8)
30–39	60 (51.3)
40–49	15 (12.8)
≥ 50	6 (5.1)
Gender	
Males	18 (15.4)
Females	99 (84.6)
Marital status	
Married	81 (69.2)
Not married^a^	36 (30.8)
Nursing qualification	
Diploma	103 (88.0)
Degree	14 (12.0)
Working experience as a nurse (years)	
≤ 5	41 (35.0)
> 5	76 (65.0)
Postgraduate ergonomic training	
Yes	50 (42.7)
No	67 (57.3)
Area of work	
Medical wards	54 (46.2)
Maternity wards	46 (39.3)
Surgical wards	7 (6.0)
Other wards^b^	10 (8.5)
Ward rotations	
Rotated	66 (56.4)
Not rotated	51 (43.6)
Have another job	
Yes	25 (21.4)
No	92 (78.6)
First episode of WMSDs^c^	
Before training as a nurse	4 (3.6)
As a student nurse	35 (31.2)
First 5 years of working	59 (52.7)
Above 5 years	14 (12.5)

^a^Not married meant not staying with a man or woman at the time of the study either single, widowed or divorced

^b^Critical units i.e. intensive care unit (ICU), coronary care unit CCU, burns unit; theatre unit; outpatient department; casualty department

^c^Calculated out of 112 who indicated for lifetime prevalence of WMSDs

The “lifetime prevalence” of WMSDs among nurses was 95.7% (n = 112). The first episodes of WMSDs were experienced in the first 5 years of working by most nurses (n = 59, 52.7%). However, 96 (82.1%) nurses reported WMSDs in the last 12-months. There was no significant difference in the 12-month prevalence of WMSDs by gender [X^2^(1) = 3.42, p = 0.06] (Table 2). The 12-month prevalence was associated with repeatedly performing nursing tasks (p = 0.005), perceptions of treating large number of patients (p = 0.004), perceptions of repeatedly bending/twisting back (p = 0.03) and perceptions of lifting/transferring dependent patients and materials (p = 0.003) (Table 3). Most nurses frequently injured the back (n = 81, 84.3%) especially the lumber region (n = 55, 67.9%). Of the 96 nurses with WMSDs, 84 (87.5%) experienced at least one of the consequences of WMSDs. Most nurses (n = 67, 79.8%) had to take a day off from work. Sixty (71.4%) nurses had to consult a health-care professional at least once in the last 12-months. However, 63 (75%) reported taking pain medication for WMSDs.

**Table 2:** Factors associated with WMSDs (N = 117)

Characteristic	Frequency	WMSD n (%)	No WMSD n (%)	Chi square	p value
Gender					
Male	18	12 (66.7)	6 (33.3)	X^2^(1) = 3.42	0.06
Female	99	84 (84.9)	15 (15.1)		
Age (years)					
20–29	36	29 (80.6)	7 (19.4)	X^2^(3) = 1.62	0.65
30–39	60	51 (85.0)	9 (15.0)		
40–49	15	11 (73.3)	4 (26.7)		
≥ 50	6	5 (83.3)	1 (16.7)		
Marital status					
Married	81	67 (82.7)	14 (17.3)	X^2^(1) = 0.08	0.78
Not married	36	29 (80.6)	7 (19.4)		
Nursing qualification					
Diploma	103	94 (91.3)	9 (8.7)	Fishers exact	0.00
Degree	14	2 (14.3)	12 (85.7)		
Postgraduate ergonomic training					
Yes	19	8 (42.1)	11 (57.9)	X^2^(1) = 24.6	0.00
No	98	88 (89.8)	10 (10.2)		
Work experience					
≤ 5 years (junior)	41	29 (70.7)	12 (29.3)	X^2^(1) = 5.49	0.02
> 5 years (senior)	76	67 (88.2)	9 (11.8)		
Having another job					
Yes	25	23 (92.0)	2 (8.00)	Fishers exact	0.12
No	92	73 (79.3)	19 (20.7)		
Area of work					
Medical ward	54	44 (81.5)	10 (18.5)	X^2^(3) = 0.48	0.92
Maternity ward	46	37 (80.4)	9 (19.6)		
Surgical ward	7	6 (85.7)	1(14.3)		
Other wards^a^	10	9 (90.0)	1 (10.0)		
Ward rotations					
Rotated	66	54 (81.8)	12 (18.2)	X^2^(1) = 0.006	0.94
Not rotated	51	42 (82.4)	9 (17.6)		

^a^Critical units i.e. intensive care unit (ICU), coronary care unit CCU, burns unit; theatre unit; outpatient department; casualty department

**Table 3:** Factors associated with WMSDs among nurses (N = 117)

Work-related tasks	Response	WMSDs n (%)	No WMSDs n (%)	Total	Chi square	p value*
Repeatedly performing certain nursing tasks	Yes	75 (88.2)	10 (11.8)	85	8.07	*0.005*
	No	21 (65.6)	11 (34.4)	32		
	Total	96	21	117		
Treating a large number of patients each day	Yes	68 (89.5)	8 (10.5)	76	8.11	*0.004*
	No	28 (68.3)	13 (31.7)	41		
	Total	96	21	117		
Not enough rest/breaks during the day	Yes	78 (82.1)	17 (17.9)	95	Fishers^a^	0.59
	No	18 (81.8)	4 (18.2)	22		
	Total	96	21	117		
Performing manual/handling techniques	Yes	36 (80.0)	9 (20.0)	45	0.21	0.65
	No	60 (83.3)	12 (16.7)	72		
	Total	96	21	117		
Working in awkward or cramped positions	Yes	65 (82.3)	14 (17.3)	79	0.01	0.93
	No	31 (81.6)	7 (18.4)	38		
	Total	96	21	117		
Working in the same position for long periods	Yes	78 (84.8)	14 (15.2)	92	2.18	0.14
	No	18 (72.0)	7 (28.0)	25		
	Total	96	21	117		
Bending or twisting your back in an awkward way	Yes	80 (86.0)	13 (14.0)	93	4.85	*0.03*
	No	16 (47.1)	8 (52.9)	34		
	Total	96	21	117		
Reaching or working away from your body	Yes	71 (82.6)	15 (17.4)	86	0.06	0.81
	No	25 (80.6)	6 (19.4)	31		
	Total	96	21	117		
Unanticipated sudden movements or falls by patients	Yes	55 (87.3)	8 (12.7)	63	2.55	0.11
	No	41 (75.9)	13 (24.1)	54		
	Total	96	21	117		
Assisting patients during walking activities	Yes	63 (84.0)	12 (16.0)	75	0.54	0.46
	No	33 (78.6)	9 (21.4)	42		
	Total	96	21	117		
Lifting or transferring dependent patients and heavy equipment and materials	Yes	69 (89.6)	8 (10.4)	77	8.74	*0.003*
	No	27 (67.5)	13 (12.5)	40		
	Total	96	21	117		
Working at or near your physical limits	Yes	77 (84.6)	14 (15.4)	91	1.83	0.18
	No	19 (73.1)	7 (26.9)	26		
	Total	96	21	117		
Overtime, irregular shift, length of workday	Yes	89 (84.0)	17 (16.0)	106	Fishers	0.11
	No	7 (63.6)	4 (36.4)	11		
	Total	96	21	117		
Lack of assistive devices and equipment	Yes	84 (82.4)	18 (17.6)	102	Fishers	0.53
	No	12 (80.0)	3 (20.0)	15		
	Total	96	21	117		
Malfunction of equipment e.g. beds that cannot be adjusted	Yes	64 (79.0)	17 (21.0)	81	Fishers	0.72
	No	32 (88.9)	4 (11.1)	36		
	Total	96	21	117		

Significant values are in italics (p < 0.05)

* Actual p values calculated

^a^Fishers represents Fishers exact test

## Discussion

This study represents the first attempt to report on the prevalence of WMSDs among nurses in the country. This novelty renders comparison with local studies difficult as they targeted different occupational groups [19, 29, 30]. Nevertheless, innumerable studies have been conducted on WMSDs among nurses worldwide [7, 11]. The present study had relatively young nurses with the majority being females. The fact that the most participants were females was expected and reflects the gender distribution in the target population in the country. This finding is consistent with other findings [7, 11]. Most of nurses had attained a diploma as their highest qualification and had been working for < 5 years. Almost all public hospitals in Zimbabwe train “diploma” nurses against one university training “degree” nurses. This largely accounts for the preponderance of diploma nurses in the study sample. The fact the majority of the nurses had < 5 years of working experience is unclear but may suggest high turnover rates in nurses as reported elsewhere [31]. It is also possible that more experienced nurses at PGH had left in search of better opportunities or had changed duties to administrative or cleric.

This present study found that WMSDs are prevalent among nurses at PGH. These findings highlight an important and untapped occupational health issue at PGH. In addition, these findings provide support on calls on public health authorities to pay more attention on MSDs [32, 33]. These results are consistent with other studies [7, 10, 13, 15, 33–35]. The possible explanation for high prevalence of WMSDs in nurses at PGH is unclear. However, similar studies link the high prevalence among nurses to the physical nature of the job [7, 34–36]. The present study showed that perceived work-related factors such as performing nursing procedures repeatedly, treating large number of patients, bending/twisting the back and lifting/transferring of patients and equipment were associated with WMSDs among the nurses at PGH.

The present study found that a sizeable proportion of nurses experienced the first episodes of WMSDs as student nurses. The majority, however, reported the first episode during the first 5 years of working. These findings are consistent with results of Tinubu et al. [7] and possibly indicate that the onset of WMSDs is indiscriminate from training to working years. This raises serious concerns. Also, the fact that most nurses experience first episode after qualifying calls for a need to evaluate working environment in an attempt to identify the possible work-related risk factors contributing to the development of WMSDs.

The present study showed that the 12-month prevalence of WMSDs among nurses at PGH was significantly associated with factors such as working experience, nursing qualification attained, and post-graduate ergonomic training. Surprisingly though, nurses with 5 or more years of working experience had WMSDs compared to those with less. These findings are consistent with findings of Tinubu et al. [7] but contradict others [15]. Yasobant and Rajikumar [15] postulated that junior workers are highly vulnerable to WMSDs because of the vigorous working style as compared to senior nurses. However, the cumulative effects of repeated exposure to risk factors and complacence on adhering to safe working principles could possibly explain the higher prevalence in senior nurses in the present study.

The present study found that eight out of ten nurses with WMSDs reported back pain. Specifically, low back pain (LBP) was most prevalent. It may be because of bending/twisting the back in awkward ways, standing long periods when treating large number of patients, inadequate breaks and lifting/transferring dependent patients. These were the most perceived work-related factors identified by the nurses in the present study. These findings support established findings that LBP is the most prevalent MSDs in adults [17]. Also, cross-sectional studies investigating WMSDs among nurses reported consistent results [7, 10, 11, 37, 38]. Sheikhzadeh et al. [37] reported a LBP prevalence of 84% among US perioperative nurses. Fabunmi et al. [38] reported similar results among Nigerian nurses.

The present study also elicited consequences experienced by the nurses at PGH secondary to WMSDs. Approximately, nine out of ten nurses had at least one consequence to report. This shows that WMSDs are highly consequential among nurses. Other studies reported similar results [7, 39]. This finding calls for awareness programs on the existence of the problem at PGH to the nurses. The consequences involved taking a day off from work to allow for seeking of medical or non-medical treatment for the troubling symptoms. This caused 71.4% of the nurses to consult a health practitioner at least once in the last 12 months resulting in getting medication for the symptoms.

In conclusion, the present study showed that WMSDs are prevalent and consequential among nurses at PGH. However, further studies with large sample sizes conducted at various hospitals investigating the prevalence, consequences and associated factors are warranted to better inform the situation on WMSDs among nurses in Zimbabwe.

## Limitations

The results of this study should be interpreted cognisant of a number of limitations.

- The study had a low response rate compared to similar studies [7, 10–12, 20]. It is possible that non-participation bias could have influenced the observed results. Although frantic efforts were made to maximise the response rate, the low response rate was a methodological issue with the majority of the nurses failing to return the questionnaires despite several reminders. Therefore, this study should be considered as a pilot study documenting baseline findings on prevalence, consequences and work-related factors among PGH nurses in Zimbabwe.
- The study was purposively conducted at one public hospital in Harare, Zimbabwe. This affects generalisability of the results to nurses outside PGH.
- The cross-sectional nature of the study precludes deducing a cause-and-effect relationship [40]. In addition, reliance on self-reported data may engender inaccurate prevalence figures due effects of memory decay, forward telescoping and “social desirability” [12, 41].
- This study only investigated socio-demographic and work-related factors associated with WMSDs neglecting other factors that possibly may influence development of the condition.

## Additional file

**Additional file 1.** Work-related musculoskeletal disorder instrument.

## Abbreviations

CCU: coronary care unit
CI: confidence interval
ICU: intensive care unit
IQR: interquartile range
k: kappa coefficient
LBP: low back pain
MSDs: musculoskeletal disorders
NMQ: Nordic Musculoskeletal Questionnaire
PGH: Parirenyatwa Group of Hospitals
US: United States
WMSDs: work-related musculoskeletal disorders
