# Supplementary material for: Work-related musculoskeletal disorders among registered general nurses: a case of a large central hospital in Harare, Zimbabwe
Source: BMC Res Notes. 2018 May 18;11:315. doi: 10.1186/s13104-018-3412-8 (PMC5960155; doi:10.1186/s13104-018-3412-8)
Supplement: Supplementary file 1 — Additional file 1. Work-related musculoskeletal disorder instrument. [file 13104_2018_3412_MOESM1_ESM.docx]

**Questionnaire** *reference code***:………**

**Demographics and work-related information**

1. Age : years
2. Gender : Male Female
3. Marital status :

- Single
- Married
- Widowed
- Divorced

1. Level of education: (please indicate by ticking)

Diploma:

Degree (bachelors/ masters/ doctorate):

Other…………………

1. Which area does your current work involve?

Neurology Orthopaedics Maternity

Surgery Medical wards Psychiatry

Critical units ^(i.e. ICU; CCU, burns unit; theatre unit; outpatient department; casualty department)^

Other (specify)

1. Which other rotations/ wards/ areas did you cover in the last 12 months?
2. How many years of experience, from qualifying do you have?
3. For many hours roughly would you say work in a day (please indicate by ticking)

| 0-8 hours | 8+ hours |
| --- | --- |
|  |  |

1. On average, how many days do you work per week? : days

Section A: Information on work related musculoskeletal disorders

**Instructions:** *You are kindly requested to fill in the questionnaire by putting an ‘X’ in the appropriate box. One ‘X’ for each question. You may be in doubt as to how to answer some questions, but do your best anyway***.**

1. Have you ever experienced any ***ache, pain, discomfort or numbness*** in the past ***12 months*** that you experienced as result of work-related activities?

Yes No

1. In the table below, the body parts are shown. Please indicate where your pain, ache, or discomfort is located, if any.

| **Body region** |  | **Frequency of problem**  **(indicate once/ twice/ etc)** |
| --- | --- | --- |
| Neck  No | Yes |  |
| Shoulder  No | Yes : right shoulder  : left shoulder  : both shoulders |  |
| Elbows  No | Yes : right elbow  : left elbow  : both elbows |  |
| Wrists/hands  No | Ye: Yes : right side  : left side  :both sides |  |
| Upper back/ lower back  No | Yes |  |
| One or both hips/thighs  No | Yes |  |
| Knees  No | Yes : right knee  : left knee  : both knees |  |
| Ankles/feet  No | Yes |  |

Section B: Consequences of injury

- Have you ever, at any point, been ***prevented from doing your normal work*** in the past ***12 months*** because of troubles with pain or discomfort?

Yes No

Below is a list of possible consequences as a result of your work-related injury. Please indicate by ticking the measures you took following your injury.

| **Consequences** | **yes** | **no** |
| --- | --- | --- |
| Modified techniques and procedures |  |  |
| Sought treatment or consultation |  |  |
| Took prescribed medication |  |  |
| Changed duties |  |  |
| Changed work setting |  |  |
| Decreased patient contact hours |  |  |
| Changed type of patient predominantly treating |  |  |
| Took time off on sick leave |  |  |
| Sought alternative treatments |  |  |
| Exercise or posture program |  |  |
| Took time off on workers’ compensation |  |  |

Other consequences……………………………………………………………………………..

Section C: Perceived causes and work related factors

*The list below describes factors that could contribute to work related MSCs amongst nurses and rehabilitation practitioners. In your opinion, to what extent do these factors contribute to your musculoskeletal condition? (Please indicate by ticking the most applicable response).*

NB: *Please do not include any pain/discomfort/injury that was acquired outside of the setting*.

| **Risk factors** | **yes** | **no** |
| --- | --- | --- |
| Repeatedly performing nursing tasks |  |  |
| Treating a large number of patients each day |  |  |
| Not enough rest/breaks during the day |  |  |
| Performing manual nursing techniques |  |  |
| Working in awkward or cramped positions |  |  |
| Working in the same position for long periods |  |  |
| Bending or twisting your back in an awkward way |  |  |
| Reaching or working away from your body |  |  |
| Unanticipated sudden movements or falls by patients |  |  |
| Assisting patients during gait activities |  |  |
| Lifting or transferring dependent patients |  |  |
| Carrying, lifting or moving heavy materials |  |  |
| Working at or near your physical limits |  |  |
| Overtime, irregular shift, length of workday |  |  |
| Inadequate training in injury prevention |  |  |
| Lack of assistive devices and equipment |  |  |
| Malfunction of equipment e.g. beds that cannot be adjusted |  |  |
